# An interactive web application for the dissemination of human systems immunology data

**DOI:** 10.1186/s12967-015-0541-x

**Published:** 2015-06-19

**Authors:** Cate Speake, Scott Presnell, Kelly Domico, Brad Zeitner, Anna Bjork, David Anderson, Michael J. Mason, Elizabeth Whalen, Olivia Vargas, Dimitry Popov, Darawan Rinchai, Noemie Jourde-Chiche, Laurent Chiche, Charlie Quinn, Damien Chaussabel

**Affiliations:** Benaroya Research Institute, Systems Immunology Laboratory, 1201 Ninth Ave., Seattle, WA 98101 USA; Sidra Medical and Research Center, Doha, Qatar; Aix-Marseille University, Marseille, France; Department of Internal Medicine and Infectious Diseases, European Hospital, Marseille, France

**Keywords:** Transcriptomics, Software, Immunology, Bioinformatics

## Abstract

**Background:**

Systems immunology approaches have proven invaluable in translational research settings. The current rate at which large-scale datasets are generated presents unique challenges and opportunities. Mining aggregates of these datasets could accelerate the pace of discovery, but new solutions are needed to integrate the heterogeneous data types with the contextual information that is necessary for interpretation. In addition, enabling tools and technologies facilitating investigators’ interaction with large-scale datasets must be developed in order to promote insight and foster knowledge discovery.

**Methods:**

State of the art application programming was employed to develop an interactive web application for browsing and visualizing large and complex datasets. A collection of human immune transcriptome datasets were loaded alongside contextual information about the samples.

**Results:**

We provide a resource enabling interactive query and navigation of transcriptome datasets relevant to human immunology research. Detailed information about studies and samples are displayed dynamically; if desired the associated data can be downloaded. Custom interactive visualizations of the data can be shared via email or social media. This application can be used to browse context-rich systems-scale data within and across systems immunology studies. This resource is publicly available online at [Gene Expression Browser Landing Page (https://gxb.benaroyaresearch.org/dm3/landing.gsp)]. The source code is also available openly [Gene Expression Browser Source Code (https://github.com/BenaroyaResearch/gxbrowser)].

**Conclusions:**

We have developed a data browsing and visualization application capable of navigating increasingly large and complex datasets generated in the context of immunological studies. This intuitive tool ensures that, whether taken individually or as a whole, such datasets generated at great effort and expense remain interpretable and a ready source of insight for years to come.

## Background

Systems studies rely on high throughput profiling technologies to measure the abundance or activity of all the constituents of a given biological system. This unbiased approach provides a global perspective on biological phenomena that can thus be studied as a whole, rather than a sum of parts. It has also proven a particularly powerful approach for hypothesis generation. Whole transcriptome profiling technologies constitute a robust yet affordable means to generate data on a systems scale and have been extensively used. As a result, vast amounts of transcriptome data are now available in public repositories. For example, more than 37,000 microarray or RNAseq studies are available in the NCBI Gene Expression Omnibus (GEO) repository [1], corresponding to more than 800,000 individual transcriptome profiles. In the immunology field, transcriptome profiling has allowed in-depth phenotyping of cell populations [2], the identification of transcriptional programs regulating hematopoiesis [3], lymphocyte differentiation [3, 4], and host responses [5, 6]. The use of large scale profiling technologies has transformed our understanding of human immunology [7], unraveling the novel pathways that underlie disease pathogenesis [8–10] and vaccine responses [11–13]. The data associated with each study, which tend to be underutilized beyond publication of primary results, potentially constitutes an invaluable resource when reinterpreted alongside other related datasets. It can provide context for the interpretation of newly generated data, and when analyzed collectively can yield insights that could not otherwise be obtained from the analysis of individual datasets.

We have developed an interactive data browsing application to promote the integration and dissemination of immunologically relevant transcriptome data. Rich contextual information is provided to support data interpretation and foster novel immunological insights. The Gene Expression Browser (GXB) platform leverages social media such as Google+ to provide users with the ability to gather, prioritize, and share findings that arise while browsing large scale profiling data. Such a framework was used previously to promote the dissemination of clinical and transcriptomic data generated in the course of a systems immunology study, thereby promoting exploration and discovery of novel knowledge by readers as well as increasing transparency of the data included in this publication [13]. Here we make a compendium of curated public domain datasets accessible via a web portal as a resource for the community, at [14]. In addition the source code for this software is released and made available for reuse by others at [15].

## Methods

The web application supporting the sample set storage was written in Grails [16], a web application extension of the Groovy programming language [17]. Jquery [18] and Asynchronous JavaScript and XML (AJAX) technologies were used to provide dynamic responses to user driven changes in the interface views. The web application runs in an Apache Tomcat application server [19], under the Ubuntu Linux operating system. MySQL [20] is used for the relational database back-end storage, and mongo [21] is used for NoSQL style data management where flexible schema are required.

The web application supports the importation of GEO data from .soft and .family style files, as well as Illumina BeadStudio standard output format. Authenticated users may upload data and add annotations using standard spreadsheet tools via comma-separated value (.csv) files.

Microarray chip probe mapping definitions were downloaded from Affymetrix and Illumina user support websites, and imported into the web application. In all, twenty different microarray chip types are currently supported by the application.

The R statistical computational language [22] was used in conjunction with the limma [23] package to calculate the rank order of genes based on their differential expression patterns (“rank lists”).

Source code and instructions are available at [15]. Necessary R scripts are available at [24], and starter databases referenced in the GitHub instructions are available at [25].

## Results and discussion

### Assembly of a collection of curated datasets

We have assembled and curated a collection of 169 datasets that are relevant to human immunology, representing a total of 13,089 unique transcriptome profiles. These sets were selected from studies currently available in NCBI’s Gene Expression Omnibus (GEO) [26]. We queried GEO for all datasets related to any of the following search terms: “monocyte”, “neutrophil”, “CD4”, “CD8”, “B cell”, “NK”, “Natural Killer”, “Plasma cell”, “CD19”, and “CD20”. The query list was filtered to select microarray datasets generated from human samples, with a sample count greater than 10. The selection was further refined to include datasets generated using Affymetrix or Illumina chips, the two most commonly used commercial microarray platforms; a few datasets from other platforms were also incorporated. In addition, relevance to human immunology research was assessed for each sampleset. Datasets were removed from the tool if they could not be appropriately displayed; most commonly, this happened when authors deposited normalized data into GEO but did not provide information on the type of normalization performed, which resulted in a very limited range of values detected in the dataset. Log2 normalization, which is applied to the majority of Affymetrix data, is automatically detected and addressed at loading. Separately, we queried GEO for whole-blood microarray datasets containing both disease case and control samples. The whole-blood data and cell-type specific data were all loaded into the web tool. Annotation data supplied with each dataset in GEO are stored in the tool. Annotations available from GEO sometimes provide substantial contextual information [9, 13, 27], but in most cases annotations are quite limited [28]. When available, additional annotation data found in the dataset’s primary publication were manually added to the annotation information provided with the microarray data.

**Figure 1:**
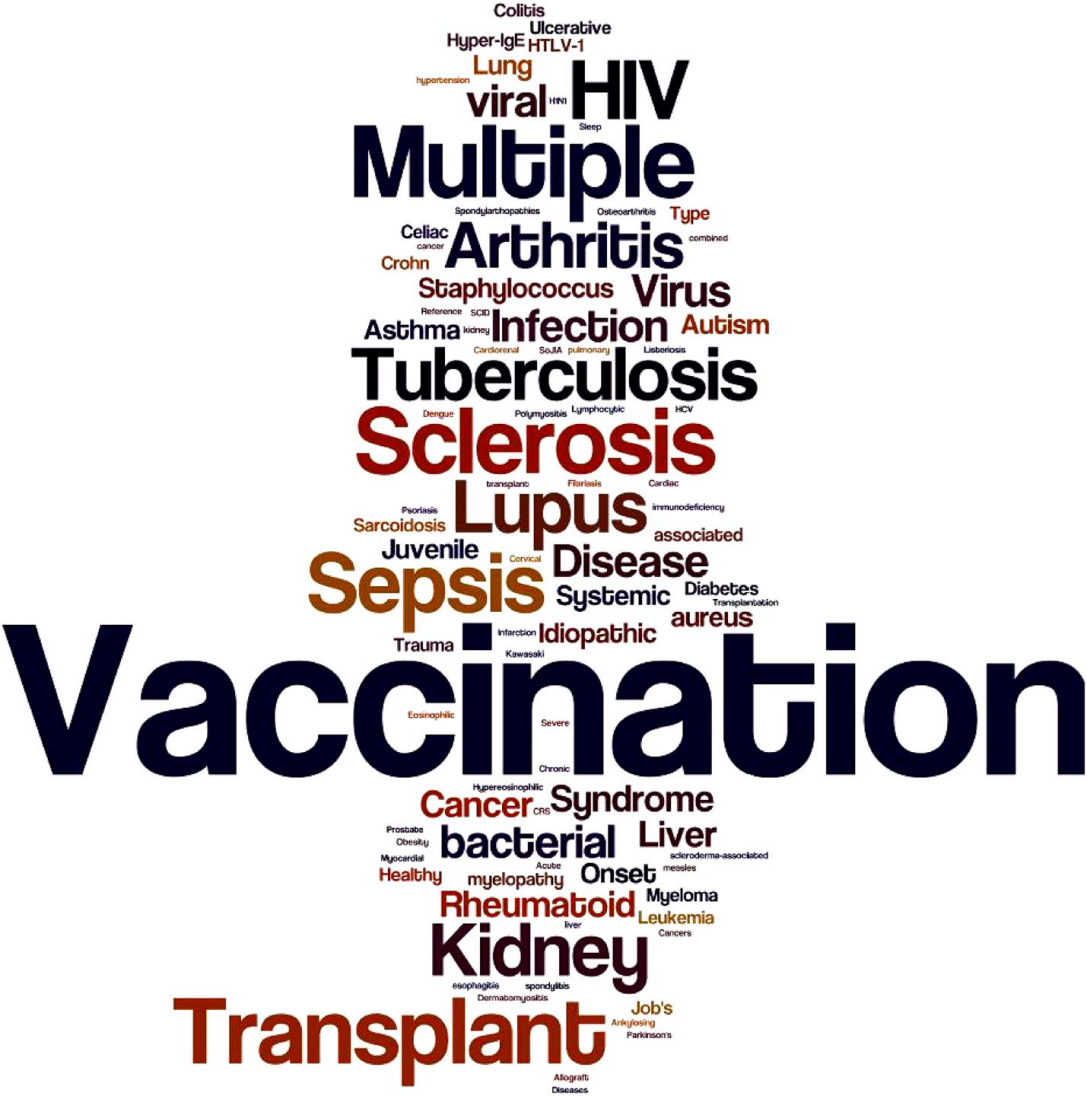
Thematic composition of the dataset collection. Word frequencies extracted from text descriptions of the studies loaded into the GXB tool are depicted as a word cloud. The size of the word is proportional to its frequency.

The datasets cover a broad swath of human immunology studies, and encompass many cell populations, tissues, diseases, and study types as illustrated by a graphical representation of relative occurrences of terms in the list of diseases loaded into our tool (Figure 1). A wide range of cell types and tissues are represented. The collection includes in vitro as well as in vivo transcriptome profiling studies. Diseases in this collection include both autoimmune and infectious diseases, as well as cancer and pathologies of the heart, brain, and blood. This collection is comprehensive but not exhaustive; users can request specific studies be added to this tool via the envelope icon at the top of the main screen. Users are encouraged to suggest GEO datasets that could be uploaded. Users can also click the envelope icon to describe private Illumina or Affymetrix human microarray datasets that they would like to make public via this tool; a member of the GXB team will contact the user to discuss.

### Data upload and processing

**Figure 2:**
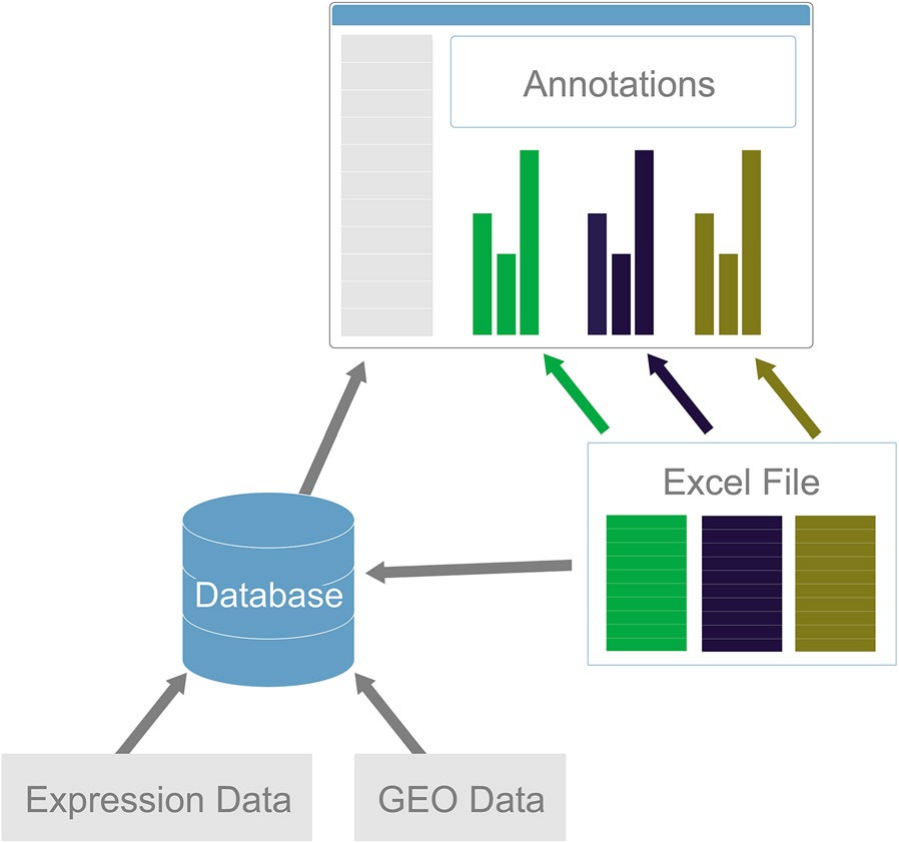
Data upload and processing. GXB takes as input both a data file of expression values and a spreadsheet of associated annotation data. It integrates these data points and displays both in a single data viewer.

Data can be imported to GXB from GEO or from lab-derived data (Figure 2). In either case, both expression values and associated annotation data are uploaded by the user. The libraries or samples identified in the first row of the data file must match the sample names in the first column of the annotation spreadsheet so that annotations can be automatically associated with samples. One of the specific strengths of GXB is the ability to take any number of annotations (categorical or numerical) for a given sample set without requiring an update of system database design; GXB is flexible to any and all data that can be associated with the samples. Importing GEO datasets reads both the expression data and annotations from either soft or series matrix formatted files. The annotations from lab-derived or GEO data import can be updated in bulk by uploading a new spreadsheet or via a web interface at any time after data upload is complete.

### Dataset navigation interface

**Figure 3:**
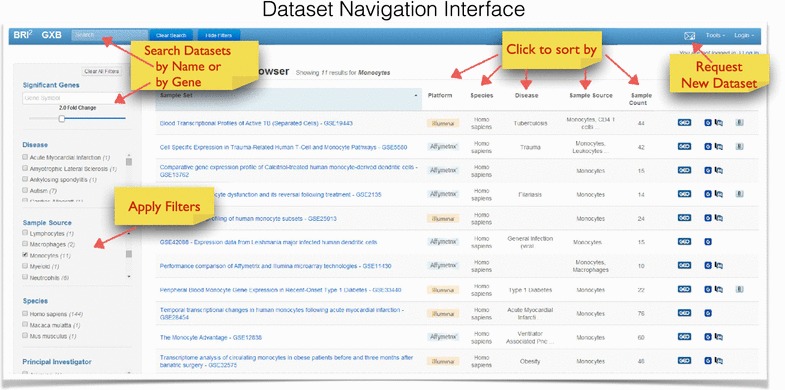
Dataset navigation interface. This interface is used for browsing, querying and filtering available datasets loaded onto the application. *Query boxes* enable search by keyword in title or by gene expression cutoff. *Check boxes* can be used for filtering. Datasets listed in the *right panel* are also sortable by each column, including Platform, Species, Disease, Sample Source, Sample Count.

The first step in transcriptional data access required development of a means to easily navigate and filter the full compendium of datasets. The “Go to GXB” button on the landing page at [14] takes users to a searchable and sortable list of datasets. Datasets of interest can be quickly identified either by filtering on criteria from pre-defined lists on the left, or by entering a query term in the search box at the top of the page (Figure 3). By default, all available datasets are listed and sorted by title. Filters are applied to produce a list of datasets that meet specific criteria: for example, choosing “tuberculosis” in the Diseases filter box returns a short list of eight datasets that include samples from TB patients. Selecting “monocytes” under the Sample Source box returns a different set of 13 datasets, profiling monocytes across several disease types. The table can also be sorted by other column headers such as platform, species, disease, sample source, and sample count. On the right of this list are icons that will take a user directly to the original dataset entry on NCBI’s GEO (G symbol) [26], or to the linked PubMed article (M symbol). The paper clip icon will take the user to a list of associated files that are available for download directly from GXB.

The database can also be queried for studies in which a gene of interest is differentially expressed between two groups. Entering an official gene symbol in the query box on the top left corner of the page will return a list of studies for which the queried gene meets a user-specified fold change cut-off. For instance, a query for IFITM3 with a twofold difference between groups returns 43 datasets. These include several studies of vaccine responses, as well as a TB dataset. It should be noted that many datasets include more than two study groups, therefore multiple comparisons are performed for any given gene. For simplicity, datasets are listed only once even though further examination may reveal that differences meeting the pre-specified cutoff can be found for more than one group comparison.

Thus, a navigation interface with advanced query and filter capabilities has been implemented to provide users with the ability to quickly identify relevant datasets for further in-depth exploration and data browsing. While the interface is intended to be easy for users to quickly identify datasets of interest, we recognize that any new software tool can be complex. A video tutorial is available as a companion to the website [29], which covers the majority of functions described in this manuscript.

### Data browsing and visualization interface

Clicking on one of the studies listed in the dataset navigation interface opens a viewer designed to provide interactive browsing and graphic representations of large-scale data in an interpretable format. This interface is designed to navigate ranked gene lists and display expression results graphically in a context-rich environment. Selecting a gene from the rank ordered list on the left will display its expression values graphically in the screen’s central panel.

**Figure 4:**
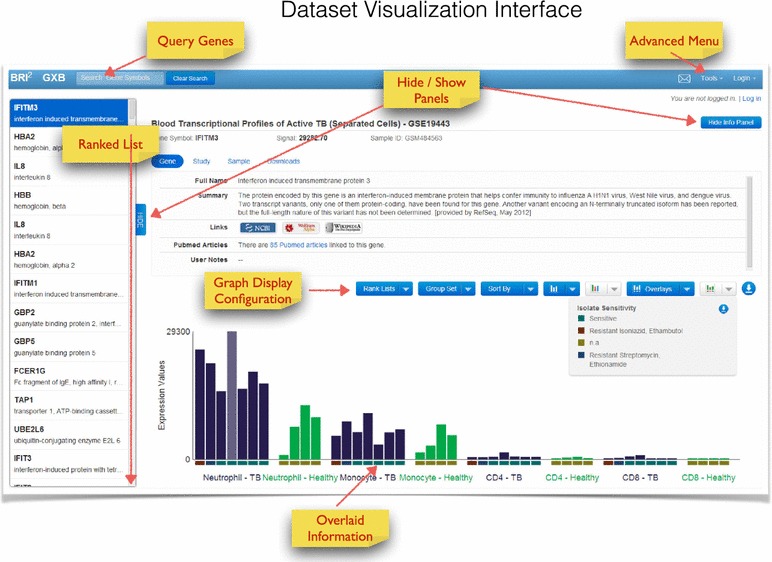
Dataset visualization interface. This interface is used for browsing, querying and displaying data in an interactive format. A scrollable ranked gene list is shown in the *left panel*. A *query box* enables search by gene symbol. Data is displayed graphically in the *right panel*. Numerous options are available for custom configuration of the graph. Contextual information can be accessed via information tabs and overlaid directly on the graph via *colored rectangles* directly below each *bar*.

Ranked ordered lists (left panel): The gene list shown on the left panel is ranked by default based on a combination of fold change and expression level difference between two groups of samples. The ranked gene list includes all the probes on an array, or in the case of RNAseq datasets all Ensemble IDs. In our example [9], genes with the greatest expression level difference between neutrophils from patients with active TB and neutrophils from healthy controls are displayed in rank order (Figure 4). Gene symbol query results are also returned in this panel. The query box is situated in the top left corner. Queries can return exact matches (e.g. TNFSF14) as well as partial matches (e.g. TNFSF will return all members of this gene family with TNFSF in the official gene symbol).

Graphical representation (central panel): Expression values for the selected gene can be represented as a histogram, where each available sample is shown as a bar (Figure 4), or as a box plot where each sample is shown as a dot. Directly above the graphical display, drop down menus give users the ability: (a) To change how the gene list is ranked. This allows the user to change the method used to rank the genes, or to include only genes that are selected for specific biological interest. Gene lists come from the KEGG database [30], or are constituted of immune-relevant genes (e.g. cytokine ligands and receptors, T cell signaling), or of genes associated with known disease signatures (GVHD and SLE, among others). (b) To change sample groups (Group Set button). In some datasets, a user can switch between groups based on cell type to groups based on disease type, for example. (c) To sort individual samples within a group based on associated categorical or continuous variables (e.g. gender or age). (d) To toggle between the histogram view and a box plot view. Samples are split into the same groups whether displayed as a histogram or box plot. (e) To view a color legend for the sample groups. (f) To select categorical information that is to be overlaid at the bottom of the graph. For example, the user can display gender or smoking status in this manner. (g) To view a color legend for the categorical information overlaid at the bottom of the graph. (h) To download the graph as a jpeg image. After the graph has been customized it can be downloaded as seen on screen, and an advanced menu gives the user the opportunity to provide a title for the graph and change the legends for the X and Y axes.

### Data interpretation

**Figure 5:**
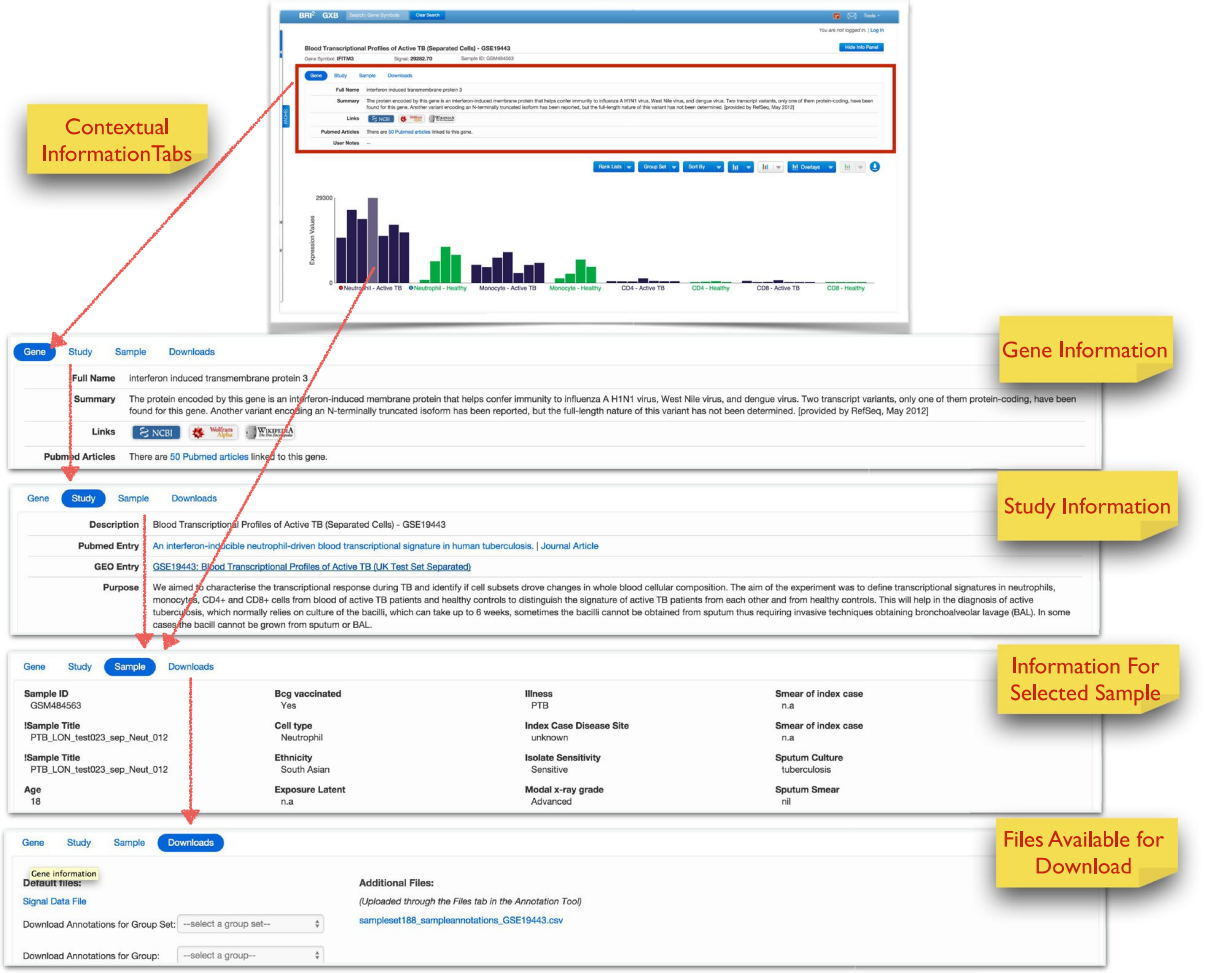
Displaying available contextual information. Contextual information available via the Gene, Study, Sample and Download tabs is shown here.

Measurements have no intrinsic utility in the absence of contextual information. It is this contextual information that makes the results of a study or experiment interpretable. It is therefore important to capture, integrate and display information that will give users the ability to interpret data and gain new insights from it. We have organized this information in tabs directly above the graphical display (Figure 5). The tabs can be hidden to make more room for the data plot display, or revealed with the blue “show info panel” button on the top right corner of the display.

Information about the gene selected from the list on the left side of the display is available under the “Gene” tab. Description of the gene function is parsed from the RefSeq database [31]. Links to the gene’s page on external resources at NCBI Gene [32], Wikipedia [33], and Wolfram Alpha [34] are also provided. A list of titles for the most recent 25 PubMed articles mentioning the gene is available with a single click, and the titles link out to PubMed for quick access to relevant literature.

Information about the study is available under the “Study” tab. Data interpretation requires an understanding of how the study or experiment was done and why it was done. This section provides background information about the study design, a reference to the primary publication associated with the dataset, and a link to the PubMed abstract.

Information available about individual samples is provided under the “Sample” tab. Rolling the mouse cursor over a histogram bar while displaying the “Sample” tab lists any clinical, demographic, or laboratory information available for the selected sample. When a large amount of sample information is available it can be broken down over multiple tabs, in order to display all ancillary data associated with a sample. This level of detail is rarely available for studies published in GEO, but we made use of the software application to display such information in a recent publication [13] and associated website [35].

Most of this sample information can be overlaid on the gene expression data histogram as colored rectangles at the bottom of each bar for categorical variables (Figure 4), or as data points plotted on a separate axis (see [35]). In our example from the tuberculosis study, this includes demographic data like age, gender, and ethnicity, as well as clinical and laboratory data such as radiographic extent of disease and drug resistance patterns of the infecting bacterial strains. As mentioned above, clinical and demographic data can be used to sort samples within a group. This feature makes it easy for users to quickly visualize relationships between clinical variables and changes in expression data. The customized expression data can be downloaded along with user-defined clinical overlays as publication/presentation-quality graphics with one click. Legends for both the histogram and the clinical data overlays are viewable within the tool and are downloadable as well.

Finally, the “Downloads” tab allows advanced users to retrieve the original dataset for analysis outside this tool. It also provides all available sample annotation data for use alongside the expression data in third party analysis software.

Thus this tool not only allows the navigation and querying of vast amounts of data with minimal user learning time, it is also capable of integrating heterogeneous ancillary information that is paramount for the interpretation of transcriptome data and generation of novel knowledge.

### Knowledge sharing

The envelope icon on the top right corner of the display will setup a new e-mail message in the user's default e-mail application. The e-mail is pre-populated with a web link that can load the user’s current view of the dataset, including sample groupings, information overlays, and other plot options.

**Figure 6:**
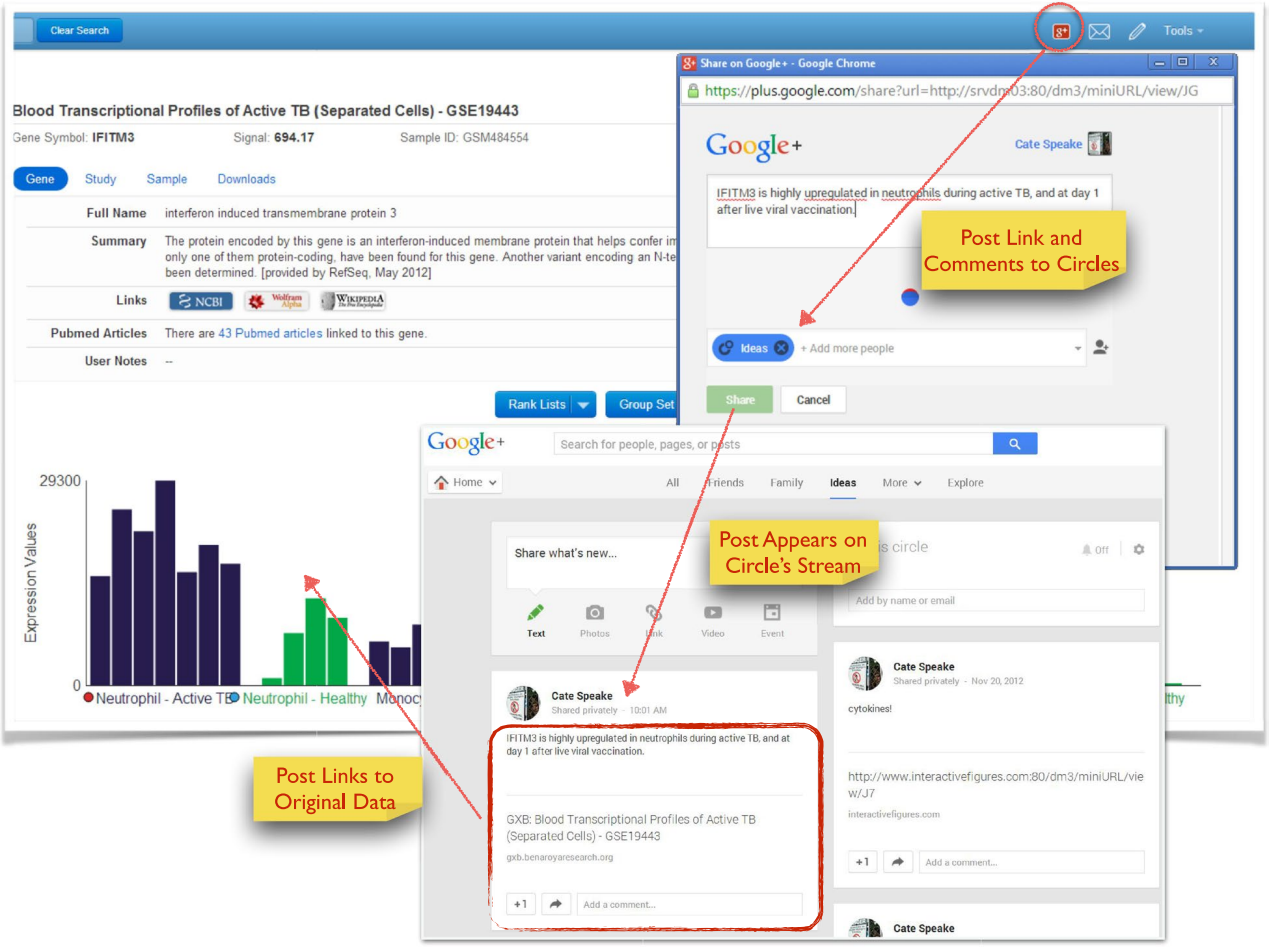
Use of social media for information archiving and sharing. A *red* Google+ button is available on the *top* navigation *bar*. Clicking this button allows users who logged into their Google+ account to feed GXB links and notes to their Google+ *circles* directly from the application. These posts can be shared publicly or with members of a *circle*. The user can also choose to keep findings private and thus use Google+ as an electronic laboratory notebook.

Systems-scale datasets provide investigators with a vast source of preliminary data that can lead to new hypotheses and new discoveries. In addition to offering ease of use and rich contextual information that facilitates data interpretation, our tool provides users with the ability to gather, organize and share findings via the social media site Google Plus (G+). The G+ link available within the tool allows users to easily and quickly archive and organize gene expression profiles of interest. Registered Google users can setup their own G+ circles (groups) and post gene expression data of interest alongside an image of that data and a weblink. A G+ circle can be empty and constitute a private archive (Figure 6). It can contain members of a laboratory or any number of colleagues, to allow lab members to share findings in real-time. A G+ circle can also be public, enabling the user to share findings with the scientific community at large. Organizing and sharing of data can also be done using email. The links shared via G+ or e-mail retain user customizations of the data view, including sample ordering and overlay of ancillary data.

## Conclusions

The web application presented here employs state of the art web programming to uniquely address Big Data’s canonical “3 Vs”, which are: Volume, Variety and Velocity [36]. Increasingly larger *V*olumes of data are generated through widespread use of systems or large-scale profiling approaches. The storage and management of such large volumes of data has become a challenge, but the GXB tool has been designed specifically to handle the large collections of datasets generated through systems-scale profiling approaches. The second V stands for *V*ariety. Datasets have become increasingly heterogeneous, especially in the context of clinical studies where a wide array of information about study subjects is available and needs to be captured. Here we have shown that GXB can incorporate and present to the user large amounts of heterogeneous ancillary information necessary for data interpretation. There is also an obvious need to integrate data generated across studies. This need will continue to grow as more clinical research studies begin to employ large scale profiling technologies in concert (e.g. genome, transcriptome, microbiome), thus heralding the era of so called “multi-omics” approaches. The third V stands for *V*elocity. Acquisition, storage, and integration of vast amounts of data is an important goal but in order to prove useful, especially as a source of novel insight and knowledge, this data must be readily and seamlessly accessible to the user. A primary strength of the GXB tool is its ability to enable rapid querying, access, and visualization of large and heterogeneous datasets. Tackling big data in the biomedical sciences will undeniably require the continued development and use of sophisticated data mining solutions, which enable bioinformaticians to map relationships in systems data and thereby reduce its dimensions [37]. It will also require the development of tools like the GXB that can engage the participation of the biomedical research community at large, can expose knowledge gaps, and can hopefully accelerate the pace of medical discoveries.

While other data viewers share some characteristics with the GXB [2, 38–45], none have fully integrated all attributes available into a tool that is designed to address the challenges posed by biomedical big data. Special emphasis has been put into user interface design; the GXB interface is as clean and simple as possible so that the vast amount of data appears clear and seamless to each scientific user. As demonstrated in one of our earlier publications, such interactive data visualization tools can be employed for the generation of interactive supplements to static figures in publications [13]. This enables more democratic access to the data underlying each static figure, and creates the opportunity for others to derive additional insight from this vast dataset. Greater transparency is an added benefit to providing data via a web-based tool. This is especially important when publishing studies based on large-scale datasets, since those data are seldom easily visualized by reviewers or by the community. For all these reasons, the use of interactive figures and data browsing software as companion to publications must be promoted and should become widespread in scientific publishing. We hope that the data browsing software tool that we have developed will further this goal.

The GXB tool is continually being improved to better enable data sharing and analysis. In the immediate future, we will begin to support upload and analysis of RNAseq, both at the gene-level and exon-level, and of high-throughput quantitative polymerase chain reaction (qPCR) datasets. It should also be noted that the tool can be used to display other data types, such as protein or cellular measurements, regardless of whether they are high dimensional. For instance, a modified version of the tool can be used to plot results from flow cytometry studies while displaying FACS images (see [13, 46]).

Taken together, the tool and resource presented here aim to democratize access to biomedical big data. This resource can be accessed online and the source code is freely available at [14, 15].
